# Clonidine prevents radiation-induced cell death in human brain organoids

**DOI:** 10.1038/s41598-025-26170-2

**Published:** 2025-10-31

**Authors:** Martin Lundberg, Samudyata, Marja Koskuvi, Asimenia Gkogka, Mahnaz Nikpour, Melis Çelik, Erik Smedler, Bo Stenerlöw, Per-Arne Lönnqvist, Martin Schalling, Carl M. Sellgren

**Affiliations:** 1 Department of Molecular Medicine and Surgery, Karolinska Institutet, Stockholm, Sweden; 2 Center for Molecular Medicine, Karolinska University Hospital, Stockholm, Sweden; 3 Department of Physiology and Pharmacology, Karolinska Institutet, Stockholm, Sweden; 4 Department of Oncology-Pathology, Karolinska Institutet, Stockholm, Sweden; 5 Department of Psychiatry and Neurochemistry, Institute of Neuroscience and Physiology, University of Gothenburg, Gothenburg, Sweden; 6 Department of Clinical Chemistry, Sahlgrenska University Hospital, Gothenburg, Sweden; 7 Wallenberg Centre for Molecular and Translational Medicine, University of Gothenburg, Gothenburg, Sweden; 8 Department of Immunology, Genetics and Pathology, Uppsala University, Uppsala, Sweden; 9 Center for Psychiatry Research, Department of Clinical Neuroscience, Karolinska Institutet & Stockholm Health Care Services, Stockholm County Council, Karolinska University Hospital, Stockholm, Sweden

**Keywords:** Brain organoids, Cell death, Clonidine, Induced pluripotent stem cells, Neuroprotection, Radiotherapy, Radiotherapy, Encephalopathy, Induced pluripotent stem cells, Neurological models, Drug discovery

## Abstract

Radiotherapy is a standard treatment of pediatric brain tumors. Though the survival rate has improved for many tumor types, most patients suffer long-term cognitive decline and there is currently no way of preventing radiation-induced damage to healthy brain tissue. Here, we used a human forebrain organoid model to investigate if the α_2_-adrenoceptor and I_1_-imidazoline receptor agonist clonidine could prevent radiotoxicity. We found that treatment of organoids with clonidine significantly reduced radiation-induced loss of neural progenitor cells, neurons, astrocytes, and oligodendrocyte lineage cells. Moreover, clonidine reduced overall DNA damage and signs of reactive gliosis in organoids. Our findings demonstrate that pharmacological rescue of radiation neurotoxicity is possible in a human brain organoid model and provides a rationale for future drug repurposing studies aiming to prevent radiation-induced brain injury in children treated with radiotherapy.

**Supplementary Information:**

The online version contains supplementary material available at 10.1038/s41598-025-26170-2.

## Introduction

Cranial irradiation is a standard treatment of pediatric brain tumors. With improvement of care, the 5-year survival rate for most tumor types now exceeds 65%.^1^ However, follow-up studies reveal that most patients, especially if they received radiotherapy at a young age, and especially if exposed to supratentorial radiation, as is often the case when treating medulloblastoma, display disabling long-term neurocognitive decline. The mechanisms behind the radiation-associated cognitive syndrome, resulting from radiotoxicity to healthy brain tissue, are poorly understood and there are currently no approved treatments to mitigate the adverse effects of radiotherapy.^3^

Previous studies have primarily focused on mechanisms of disability that appear between 6 months and 1 year after radiotherapy.^3^ However, recent studies show that central nervous system (CNS) alterations and dysfunctions develop much earlier following radiation exposure.^4^ This has prompted the hypothesis that early forms of radiation-induced CNS damage could drive chronic pathophysiological processes that lead to permanent cognitive decline.^3^ So far, our limited understanding of how early radiation-induced brain injury results in long-term cognitive disability stems from rodent models. These early radiation effects include loss of neuronal precursors, oligodendrocyte injury, reactive gliosis, neuroinflammation, and vascular endothelial damage.^3^ A few clinical studies have attempted to mitigate brain radiotoxicity but have yielded mixed and short-lasting effects.^5^

Recently, the ability to differentiate human induced pluripotent stem cells (iPSCs) into three-dimensional tissue cultures, which recapitulate several aspects of the cytoarchitecture and cell–cell interactions of the developing human brain, has emerged as a promising tool to study various aspects of human neurobiology.^6^ Such experimental model systems, typically referred to as brain organoids, can also be a useful tool in the context of drug discovery, as well as for providing a foundation for rational drug repurposing studies.

Clonidine, a mixed α_2_-adrenoceptor and I_1_-imidazoline receptor agonist, produces sedative, analgesic, anxiolytic, and antihypertensive effects through central suppression of the sympathetic nervous system and is widely used in pediatric anesthesia.^7^ In addition, clonidine has a wide range of uses in neurological and psychiatric disorders such as neonatal opioid-withdrawal syndrome, ADHD, and Tourette’s syndrome.^8^ Clonidine has also been suggested as a potential neuroprotective drug due to several studies reporting significant effects of clonidine treatment on reducing ischemic brain injury and glutamate excitotoxicity in rodent models.^9,10^.

Here, we conducted a proof-of-concept study to investigate if clonidine could prevent radiation-induced injury in a human organoid model. We found that clonidine treatment of organoids largely prevented radiation-induced cell death of neurons as well as glia, reduced signs of reactive gliosis, and decreased overall levels of DNA damage.

## Results

### Generation and characterization of human iPSC-derived forebrain organoids

**Fig. 1:**
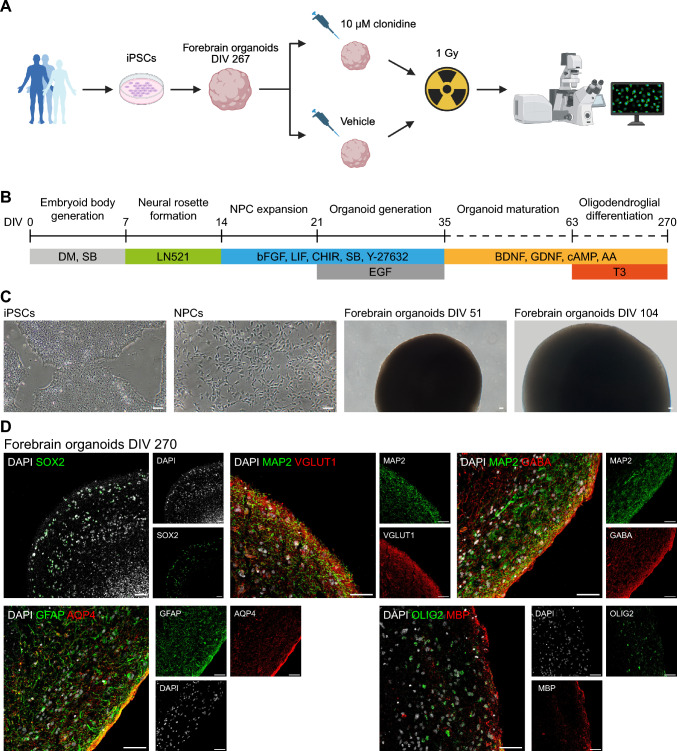
Generation and characterization of iPSC-derived forebrain organoids. (**A**) Graphical illustration of study design (created in BioRender). Forebrain organoids were generated from iPSCs from three healthy subjects. At DIV 267, the organoids were treated with either 10 µM clonidine or vehicle for 24 h before being irradiated at 1 Gy and then treated for an additional 48 h before analysis. (**B**) Forebrain organoid culture scheme. (**C**) Representative brightfield images at different stages of development. (**D**) Representative confocal images of organoid sections at DIV 270 stained for SOX2 (neural progenitor cells), MAP2 (neurons), VGLUT1 (glutamatergic neurons), GABA (GABAergic neurons), GFAP and AQP4 (astrocytes), OLIG2 (oligodendrocyte lineage cells), and MBP (myelinating oligodendrocytes). Scale bars, 50 µm.

To model radiation-induced damage to healthy human brain tissue we first generated human iPSC-derived brain organoids patterned towards the forebrain, i.e., the early brain structure that give rise to telencephalon and diencephalon in which several common pediatric brain tumors originate.^11^ First, iPSCs were derived from fibroblasts of three healthy subjects (two males and one female) via mRNA reprogramming^12^ (Fig. 1A). We then generated cerebral organoids utilizing a protocol which includes forebrain patterning and addition of factors promoting oligodendrogenesis^13,14^ (Fig. 1B–C) and cultured the organoids up to a total of 270 days in vitro (DIV). Using immunohistochemistry, we then confirmed the presence of neural progenitor cells (SOX2), neurons (MAP2) of both glutamatergic (VGLUT1) and GABAergic (GABA) identity, astrocytes (GFAP, AQP4), and oligodendrocyte lineage cells (OLIG2), including myelinating oligodendrocytes (MBP) (Fig. 1D).

### Clonidine prevents radiation-induced depletion of neuronal and glial cells and reduces levels of reactive gliosis

**Fig. 2:**
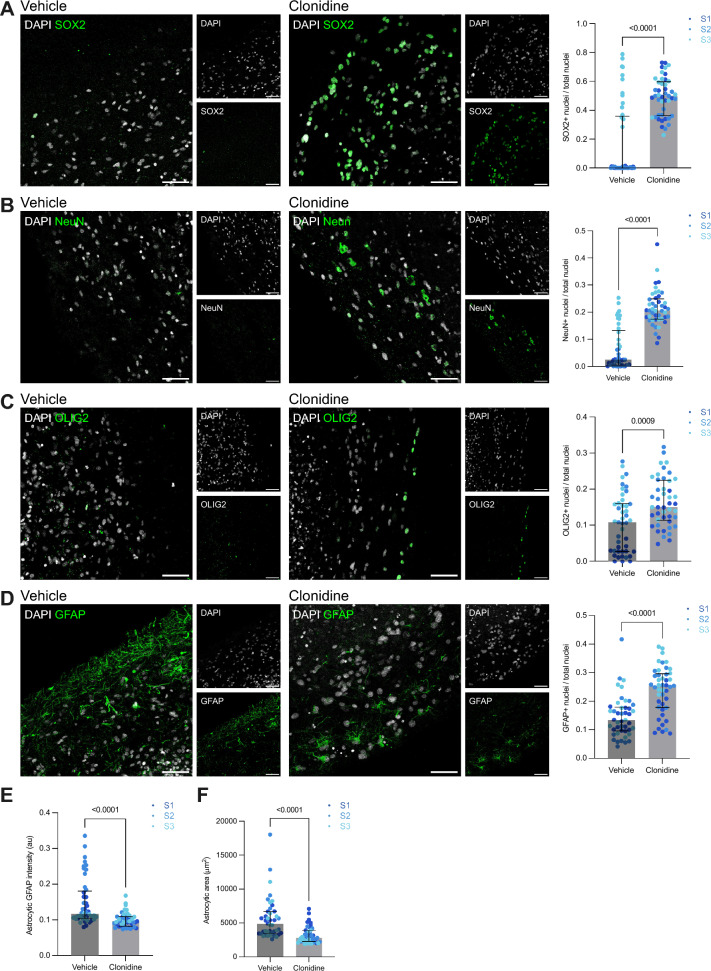
Clonidine prevents radiation-induced depletion of neuronal and glial cells and reduces reactive gliosis. Representative images and quantification of SOX2^+^ neural progenitor cells (**A**), NeuN^+^ neurons (**B**), OLIG2^+^ oligodendrocyte lineage cells (**C**), and GFAP^+^ astrocytes (**D**) in irradiated organoids treated with clonidine or vehicle and analyzed at DIV 270. Mean nuclear GFAP intensity in GFAP^+^ astrocytes (**E**) and mean astrocytic area (**F**) at DIV 270. For each of the three subjects, one organoid per condition was included. For each organoid, three sections were assayed using five images per section. Each data point corresponds to one image. Between-group differences were analyzed using Mann–Whitney *U* tests (*p* values are two-tailed). Bars represent medians and whiskers represent interquartile range. au = arbitrary units. Scale bars, 50 µm. S1, S2, and S3 denotes the three subjects included in the study.

To test whether clonidine treatment could protect against early radiation-induced neural injury we exposed the generated organoids to either 10 μM clonidine hydrochloride or water (vehicle) for 24 h before and 48 h after irradiation at a dose of 1 Gy (Fig. 1A). We found that significantly more SOX2^+^ neural progenitor cells survived irradiation in clonidine-treated organoids as compared to vehicle-treated organoids (vehicle: median (Mdn) = 0.004; interquartile range (IQR) = 0.00–0.36, clonidine: Mdn = 0.50; IQR = 0.36–0.60, Mann–Whitney *U* test: *p* < 0.0001) (Fig. 2A). Similarly, we observed a greater survival of NeuN^+^ neurons in the clonidine-treated organoids (vehicle: Mdn = 0.025; IQR = 0.006–0.13, clonidine: Mdn = 0.21; IQR: 0.17–0.25, Mann–Whitney *U* test: *p* < 0.0001) (Fig. 2B).

Previous rodent studies have also reported that cranial irradiation can lead to a loss of cycling oligodendrocytes.^15^ We therefore investigated the effect of clonidine on OLIG2^+^ oligodendrocyte lineage cells and found that clonidine also reduced radiation-induced oligodendrocyte lineage cell death (vehicle: Mdn = 0.11; IQR = 0.028–0.16, clonidine: Mdn = 0.15; IQR = 0.11–0.22, Mann–Whitney *U* test: *p* = 0.0009) (Fig. 2C). Furthermore, early effects of radiation on the CNS has also been suggested to include astrocyte damage as well as increased reactivity of astrocytes, characterized by an increased astrocytic expression of GFAP and hypertrophy of astrocytic processes.^3^ We found that more astrocytes survived radiation in the clonidine-treated organoids (vehicle: Mdn = 0.13; IQR = 0.096–0.18, clonidine: Mdn = 0.25; IQR = 0.18–0.3, Mann–Whitney *U* test: *p* < 0.0001) (Fig. 2D). Furthermore, astrocytes displayed lower levels of GFAP in the clonidine-treated organoids (vehicle: Mdn = 0.12; IQR = 0.1–0.18, clonidine: Mdn = 0.096; IQR = 0.082–0.11, Mann–Whitney *U* test: *p* < 0.0001) (Fig. 2E). The astrocytes were also smaller on average in the clonidine-treated organoids (vehicle: Mdn = 4819; IQR = 3489–6685, clonidine: Mdn = 2825; IQR = 2304–3906, Mann–Whitney* U* test: *p* < 0.0001) (Fig. 2F).

### Clonidine reduces radiation-induced DNA damage and apoptosis

**Fig. 3:**
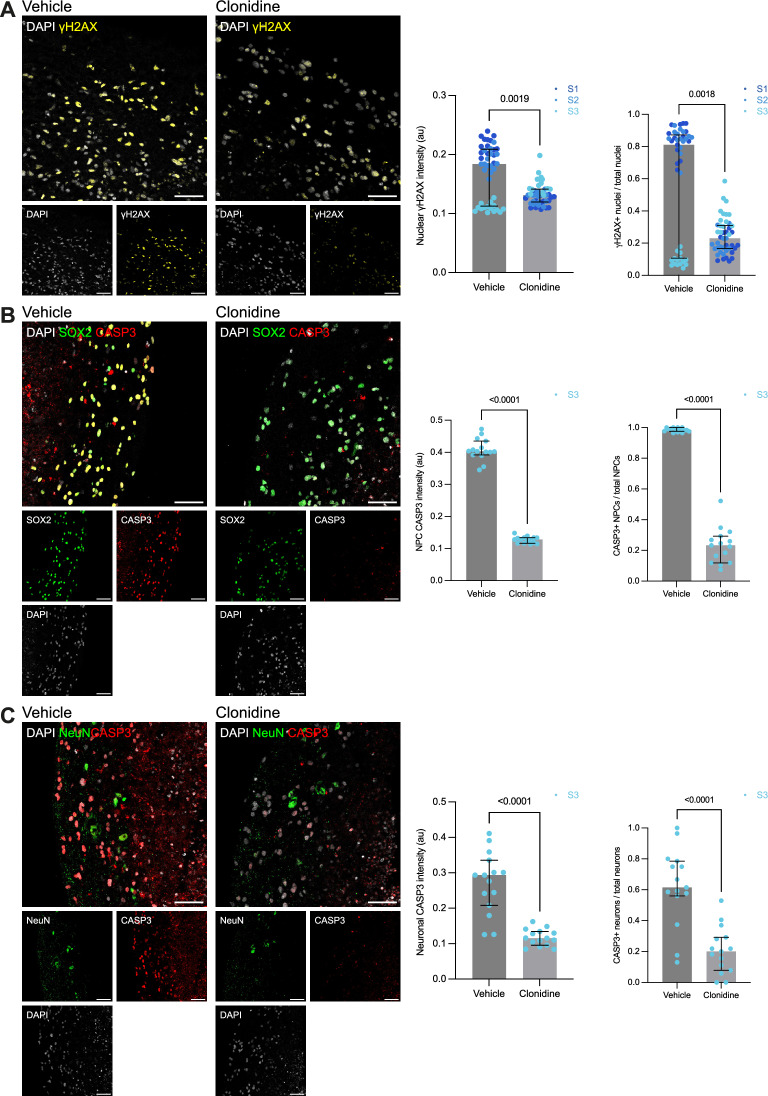
Clonidine reduces radiation-induced DNA damage and apoptosis. (**A**) Representative images and quantification of γH2AX-positive nuclei and of mean nuclear γH2AX intensity as a measure of DNA damage in irradiated organoids treated with clonidine or vehicle and analyzed at DIV 270. (**B**) Representative images and quantification of CASP3-positive nuclei and of mean nuclear CASP3 intensity in SOX2^+^ neural progenitor cells (**B**) and NeuN^+^ neurons (**C**) as measures of apoptosis in the irradiated organoids of subject S3 at DIV 270. For each of the three subjects, one organoid per condition was included. For each organoid, three sections were assayed using five images per section. Each data point corresponds to one image. Between-group differences were analyzed using Mann–Whitney *U* tests (*p*-values are two-tailed). Bars represent medians and whiskers represent interquartile range. au = arbitrary units. Scale bars, 50 µm. S1, S2, and S3 denotes the three subjects included in the study.

To explore whether clonidine also has an effect on radiation-induced DNA damage, we analyzed levels of γH2AX, a marker of double-strand DNA breaks. In clonidine-treated organoids, γH2AX levels were lower than in vehicle-treated organoids (vehicle: Mdn = 0.18; IQR 0.11–0.21, clonidine: Mdn = 0.13; IQR = 0.12–0.14, Mann–Whitney *U* test: *p* = 0.0019) (Fig. 3A), indicating that clonidine could attenuate radiation-induced DNA damage. The number of γH2AX-positive nuclei were also lower in clonidine-treated organoids (vehicle: Mdn = 0.81; IQR = 0.11–0.87, clonidine: Mdn = 0.23; IQR = 0.17–0.31, Mann–Whitney *U* test: *p* = 0.0018) (Fig. 3A). Notably, organoids generated from one of the three donors were more resilient to cell depletion (neural progenitor cells as well as neurons, see Fig. 2A and B). To determine whether clonidine still mediated a neuroprotective effect in this model, we analyzed levels of cleaved caspase-3 (CASP3), a marker of programmed cell death. The analysis revealed that cells which survived radiation in the vehicle-treated organoid were largely apoptotic and that clonidine in fact reduced the levels of apoptosis among neural progenitor cells (vehicle: Mdn = 0.4; IQR = 0.39–0.44, clonidine: Mdn = 0.13; IQR = 0.12–0.13, Mann–Whitney *U* test: *p* < 0.0001) (Fig. 3B) as well as in neurons (vehicle: Mdn = 0.29; IQR = 0.21–0.34, clonidine: Mdn = 0.12; IQR = 0.096–0.13, Mann–Whitney *U* test: *p* < 0.0001) (Fig. 3C). This was also the case when analyzing number of CASP3-positive neural progenitor cells (vehicle: Mdn = 0.98; IQR = 0.98–1, clonidine: Mdn = 0.23; IRQ = 0.12–0.29, Mann–Whitney *U* test: *p* < 0.0001) (Fig. 3B) and number of CASP3-positive neurons (vehicle: Mdn = 0.62; IQR = 0.56–0.79, clonidine: Mdn = 0.2, IQR = 0.079–0.29, Mann–Whitney *U* test *p* < 0.0001) (Fig. 3C).

## Discussion

In this study, we investigated whether clonidine, a mixed α_2_-adrenoceptor and I_1_-imidazoline receptor agonist commonly used in pediatric anesthesia, could reduce the effects of radiotoxicity in a human iPSC-derived forebrain organoid model. We found that clonidine treatment largely prevented radiation-induced cell depletion of neural progenitor cells, neurons, astrocytes, and oligodendrocyte lineage cells. Moreover, we observed reduced astrocyte reactivity and lower levels of DNA damage in the clonidine-treated organoids. Overall, our findings provide proof-of-concept that a human brain organoid model system can capture protective pharmacological effects on early radiation-induced injury.

The largest effect of clonidine in terms of preventing radiation-induced cell depletion could be observed in the neural progenitor cell and neuron populations. This is in line with previous rodent studies indicating that neural progenitor cells are particularly vulnerable to radiation damage^16^ and that neurons are more sensitive to radiation than glia^17^. Interestingly, in organoids from one of the three donors (female), the neural progenitor cells and neurons were more resilient to radiation-induced cell death than in the organoids from the other two donors (males) and largely survived even without clonidine treatment. However, subsequent analyses revealed that, even though these cells survived, they were undergoing apoptosis which was largely prevented by clonidine treatment. Though this may be a result of individual variability, previous studies have found female mice to be more resilient to radiation damage when compared to their male conterparts^18,19^, indicating the possibility of sex-specific difference in response to radiotoxicity.

While we observed that a larger number of astrocytes survived in the clonidine-treated organoids, we also observed lower astrocytic GFAP levels, as well as smaller astrocytic size, in clonidine-treated organoids, indicating that clonidine reduces astrogliosis, while simultaneously limiting astrocytic cell death. Astrocytes become reactive in response to various kinds of CNS injury, including insults caused by radiation, which leads to morphological changes, upregulation of GFAP protein levels, and eventually formation of glial scars.^20^ Lower astrocyte reactivity in clonidine-treated organoids is likely a result of an overall reduction in neural tissue damage, prompting less of a response from the astrocyte population.

The protective effect of clonidine on neuronal and glial cell death in irradiated organoids could potentially be mediated by the prevention of DNA damage, as indicated by overall lower levels of nuclear γH2AX in clonidine-treated organoids. Phosphorylation of H2AX, creating γH2AX, is the first step in recruiting DNA repair proteins to sites of double-stranded breaks.^21^ Radiation-induced DNA damage is a result of both direct effects of ionizing radiation on the DNA molecule, and secondary effects caused by the generation of reactive oxygen species. DNA-breaks triggers the recruitment of the DNA damage repair machinery which, if successful, enable cells to survive, but if unsuccessful, induces programmed cell death.^22^.

In the female line, in which irradiation caused the least amount of cell death, levels of nuclear γH2AX was also found to be lower in the vehicle condition relative to the other two lines. However, in this line, clonidine paradoxically increased levels of nuclear γH2AX even though the overall amount of the amount of apoptotic activity was reduced. This raises the question whether increased recruitment of DNA repair proteins could be part of the mechanism by which clonidine reduced levels of apoptosis in this line. There are studies indicating sex differences in cellular responses to DNA damage and a greater ability among females to mount DNA repair responses.^23^ However, to address possible sex differences, our study would have required a greater sample size of both males and females.

Our findings of neuroprotective effects of clonidine against radiation damage in human forebrain organoids is in line with previous animal studies reporting neuroprotective effects of clonidine on various sources of neural damage including ischemic brain injury and glutamate excitotoxicity.^9,10^ Several mechanisms behind the neuroprotective effect of clonidine have been suggested, including reduced sympathetic activity, decreased release of glutamate and aspartate, regulation of NMDA-receptors, regulation of mitochondrial activity, and attenuated microglial activation.^9,10,24,25^ Interestingly, we were able to observe significant neuroprotective effects of clonidine even though our organoid model did not contain either vascular endothelial cells or microglia, indicating that the effect, at least in part, is mediated by direct effects on neurons, astrocytes or oligodendroglia lineage cells. Future studies should aim to further investigate these mechanisms in a human context.

Given that clonidine is widely used, easily administered, and considered a safe choice for treating children^26^, our findings of radioprotective effects of clonidine in a human forebrain organoid model provides a rational for future drug repurposing studies aiming to prevent radiation-induced brain injury in children who receive cranial irradiation. Building on recent advances in organoid generation, incorporation of brain tumor cells^27^, microglia, and vascular structures^28^ would be important next steps in further improving the model to facilitate the transition of promising drug candidates into clinical trials. Future brain tumor organoid studies will be especially important in order to determine whether clonidine also exert radioprotective effects on neuronal and glial tumor cells or not.

Several limitations related to our model system need to be considered. Organoid models can recapitulate developmental features up to the early postnatal period.^29^ It can therefore not be excluded that the effect would have been different in neural tissue at later stages of development. Moreover, while the forebrain is an appropriate region to study considering the reported cognitive deficits and the origin of certain tumors, it would also be important to evaluate the effect in other brain regions. Additionally, given our directed protocol, our organoids did not contain either vasculature, necessary to model irradiation-induced blood–brain barrier dysfunction, or microglia, a cell type which may play a role in the adverse effects of cranial irradiation by promoting neuroinflammation.^30^ As part of this proof-of-concept study, we used a single drug concentration and radiation dose and assessed the effects of clonidine at one timepoint only. Future studies should investigate the dose–response relationship as well as the long-term effects of clonidine on preventing radiotoxicity in human brain organoids. Moreover, while we assessed neuroprotective effects in terms of cellular viability across cell types, we did not assess the effect of clonidine on the functionality of neural circuits. This should be addressed in future studies through electrophysiological analysis of brain organoids using calcium imaging or microelectrode arrays. Finally, despite the limited sample size in this study, it is noteworthy that we observed subject-dependent differences in resilience to radiation-induced neural damage. Thus, using larger sample sizes, our model could also be useful in identifying subject-specific factors influencing outcomes of cranial irradiation.

## Methods

### iPSC reprogramming

This study was performed in line with the principles of the Declaration of Helsinki. All subjects provided written informed consent before participating in the study. Approval was granted by the Regional Ethical Review Board in Stockholm, Sweden. Dermal biopsies were collected under standardized conditions from three healthy subjects (two males and one female) and fibroblast cultures were established as previously described.^31^ Fibroblasts were reprogrammed into iPSCs using modified mRNA reprogramming in a feeder-free culture system as described previously.^32^ Pluripotency of iPSC lines was evaluated by flow cytometry for the expression of surface markers SSEA4 (positive), SSEA1 (negative), and OCT4 (positive). The analysis was performed with a BD Stemflow Human and Mouse Pluripotent Stem Cell Analysis Kit (BD, Cat# 560,477), following manufacturer instructions (S1: flow cytometry SSEA4 100%, OCT4 98.6%; S2: flow cytometry SSEA4 100%, OCT4 96.3%; S3: flow cytometry SSEA4 100%, OCT4 96.5%). iPSCs were expanded in mTeSR Plus medium (STEMCELL Technologies, Cat# 100–0276) on plates coated with Geltrex (Gibco, Cat# A1413302). iPSCs were then purified using MACS with anti-TRA-1–60 MicroBeads (Miltenyi Biotec, Cat# 130–100-832). All fibroblasts and iPSCs tested negative for mycoplasma. To evaluate chromosomal stability, all iPSC lines also underwent classical karyotyping. The iPSC lines were cultured in T25 flasks coated with Biolaminin LN521 (LN521, BioLamina, Cat# LN521) until reaching 70–80% confluency, using mTeSR Plus medium (STEMCELL Technologies, Cat# 100–0276). Cells were treated with KaryoMAX Colcemid Solution (100 ng/ml, Thermo Fisher, Cat# 15,212,012) for 2 h to arrest them in metaphase. Subsequently, the cells were harvested using Versene Solution (Thermo Fisher, Cat# 15,040,066) and collected in PBS (Thermo Fisher, Cat# 10,010,023). Karyograms are presented in Supplementary Figure S1.

### Generation of NPCs

Neural progenitor cells (NPCs) were generated from the iPSCs as previously described with minor modifications.^14^ Briefly, at day in vitro (DIV) 0, 10,000 iPSCs were seeded into each well of a V-bottom ultra-low-attachment 96-well plate and cultured for 1 week into embryoid bodies (EBs) using EB medium consisting of Advanced DMEM/F12 (Gibco, Cat# 12,634,010), N-2 (1X, Gibco, Cat# 17,502,001), B27 without vitamin A (1X, Gibco, Cat# 12,587,010), dorsomorphin (DM, 1 μM, Sigma-Aldrich, Cat# P5499), and SB431542 (SB, 5 μM, Tocris Bioscience, Cat# 1614). At DIV 7, EBs were transferred to plates coated with Matrigel (Corning, Cat# 354,230) in medium consisting of Advanced DMEM/F12 (Gibco, Cat# 12,634,010), N-2 (1X, Gibco, Cat# 17,502,001) and LN521 (1 μg/ml, BioLamina, Cat# LN521) and cultured into neural rosettes for 1 week. At DIV 14, neural rosettes were manually isolated from surrounding cells and expanded for 1 additional week in NPC media, consisting of a 1:1 mixture of Neurobasal (Gibco, Cat# 21,103,049) and Advanced DMEM/F12 (Gibco, Cat# 12,634,010) supplemented with N-2 (1X, Gibco, Cat# 17,502,001), B27 without vitamin A (1X, Gibco, Cat# 12,587,010), basic fibroblast growth factor (bFGF, 20 ng/ml, PeproTech, Cat# 100-18B) leukemia inhibitory factor (LIF, 10 ng/ml, PeproTech, Cat# 300–05), CHIR99021 (CHIR, 3 μM, R&D Systems, Cat# 4423), SB (2 μM, Tocris Bioscience, Cat# 1614), and Y-27632 (10 μM, STEMCELL Technologies, Cat# 72,302).

### Forebrain organoid generation

Forebrain organoids were generated as previously described with minor modifications.^13,14^ Briefly, at DIV 21, 10,000 NPCs were seeded into each well of a V-bottom ultra-low-attachment 96-well plate and cultured for 3 days into NPC aggregates using neural proliferation (NP) media consisting of NPC media with the addition of epidermal growth factor (EGF, 20 ng/ml, R&D Systems, Cat# 236-EG). At DIV 24, NPC aggregates were transferred to flat-bottom ultra-low-attachment 24-well plates and cultured into immature forebrain organoids using NP media for an additional 11 days. Starting at DIV 27, plates were incubated on a shaker at a speed of 80 rpm. At DIV 35 organoids were transferred to flat-bottom ultra-low-attachment 6-well plates and cultured on a shaker into mature forebrain organoids using neural differentiation (ND) media consisting of a 1:1 mixture of Neurobasal (Gibco, Cat# 21,103,049) and Advanced DMEM/F12 (Gibco, Cat# 12,634,010) supplemented with N-2 (1X, Gibco, Cat# 17,502,001), B27 without vitamin A (1X, Gibco, Cat# 12,587,010), Penicillin–Streptomycin (0.5X, Gibco, Cat# 15,140,122), brain derived neurotrophic factor (BDNF, 20 ng/ml, PeproTech, Cat# 450–02), glial cell line-derived neurotrophic factor (GDNF, 20 ng/ml, PeproTech, Cat# 450–10), cyclic adenosine monophosphate (cAMP, 1 μM, Sigma-Aldrich, Cat# D0627), and ascorbic acid (200 nM, STEMCELL Technologies, Cat# 72,132). Starting from DIV 63, organoids were maintained in oligodendroglial differentiation (OL) media consisting of ND media supplemented with 3,3,5-Triiodo-L-thyronine sodium salt (T3, 10 ng/ml, Sigma-Aldrich, Cat# 28,745). For further neuronal maturation, organoids were cultured in BrainPhys medium (STEMCELL Technologies, Cat# 05,790).

### Treatment and irradiation of forebrain organoids

At DIV 267, organoids were treated with either 10 µM clonidine hydrochloride (Catapresan®, Glenwood GmbH, Munich, Germany, Cat# Vnr-118428) or water as vehicle and incubated for 24 h before being irradiated using a CIX2 X-Ray Irradiator (Xstrahl, Suwanee, GA, USA) with copper filter at a focus-to-skin distance (FSD) of 50 cm and with an energy of 195 kV, 10 mA at a dose rate of 0.840 Gy/min for 1 min and 10 s for a total received dose of 1 Gy. After irradiation, organoids were incubated with clonidine or vehicle for an additional 48 h with one media change after the first 24 h. 10 μM clonidine has previously been shown to be effective in reducing NMDA-induced cell death in primary cultures of cortical neurons from mice^10^ and is within the range of intrathecal doses used in clinical studies^33,34^. A pretreatment period of 24 h were selected to allow for adequate diffusion into brain organoids and for cellular effects of the drug to be initiated. A 48 h post-irradiation treatment period was chosen to allow for capture of radiation-induced effects on both DNA damage and apoptosis. A radiation dose of 1 Gy was selected as it approximately corresponds to the dose that a considerable part of healthy brain tissue is exposed to during cranial irradiation of pediatric brain tumors.^35^.

### Cryopreservation

Organoids were fixed in 4% paraformaldehyde (PFA, Thermo Scientific, Cat# 15,670,799) for 45 min, washed twice in PBS (Gibco, Cat# 10,010,023) for 5 min, and then incubated in 30% sucrose (w/vol, Sigma-Aldrich, Cat# S0389) in PBS (Gibco, Cat# 10,010,023) overnight at 4 °C. Fixed organoids were subsequently embedded in OCT Cryomount (Histolab, Cat# 45,830) and snap-frozen in isopropanol (Sigma-Aldrich, Cat# 9516) on dry ice and stored at -80 °C until cryosectioning. Frozen organoid blocks were then cryosectioned into 20 µm thick sections on a Cryostar™ NX70 cryostat (Epredia, Kalamanzoo, MI, USA). At least three sections were collected on each slide, representing different parts of each organoid.

### Immunohistochemistry

Organoid sections were washed with PBS (Gibco, Cat# 10,010,023) for 5 min, followed by permeabilization and blocking in blocking solution containing 0.3% Triton X-100 (VWR, Cat# 437002A) in PBS (Gibco, Cat# 10,010,023) with 3% bovine serum albumin (BSA, Tocris Bioscience, Cat# 5217) for 1 h at room temperature (RT). Sections were washed twice with PBS (Gibco, Cat# 10,010,023) for 5 min and then incubated with primary antibodies diluted in blocking solution overnight at 4 °C. Primary antibodies used in this study were rat monoclonal anti-SOX2 clone Btjce (1:100, Thermo Fisher Scientific, Cat# 14–9811-82, RRID:AB_11219471), chicken polyclonal anti-MAP2 (1:500, Abcam, Cat# ab5392; RRID:AB_2138153), rabbit polyclonal anti-VGluT1 (1:100, Abcam, Cat# ab272913, RRID:AB_3068553), rabbit polyclonal anti-GABA (1:500, Sigma-Aldrich, Cat# ab272913, RRID:AB_477652), rabbit polyclonal anti-GFAP (1:500, Agilent, Cat# GA524, RRID:AB_2811722), mouse monoclonal anti-AQP4 clone B-5 (1:100, Santa Cruz Biotechnology, Cat# sc-390488, RRID:AB_3083097), goat polyclonal anti-Olig2 (1:100, R&D Systems, Cat# AF2418, RRID:AB_3083097), mouse monoclonal anti-MBP clone 932,908 (1:50, R&D Systems, Cat# MAB42282, RRID:AB_3658268), mouse monoclonal anti-NeuN clone 1B7 (1:500, Abcam, Cat# ab104224, RRID:AB_10711040), mouse monoclonal anti-GFAP clone 2E1 (1:500, Santa Cruz Biotechnology, Cat# sc33673, RRID: AB_627673), mouse monoclonal anti-Phospho-Histone H2A.X (Ser139) clone D7T2V (1:100, Cell Signaling Technology, Cat# 80,312, RRID:AB_2799949), and rabbit polyclonal anti-Cleaved Caspase-3 (Asp175) (1:200, Cell Signaling Technology, Cat# 80,312, RRID:AB_2341188). Sections were then washed twice with PBS (Gibco, Cat# 10,010,023) for 5 min and then incubated with secondary antibodies diluted in blocking solution for 2 h at RT in the dark. Secondary antibodies used in this study were donkey polyclonal anti-mouse Alexa Flour 488 (1:500, Thermo Fisher Scientific, Cat# A-21202, RRID:AB_141607), donkey polyclonal anti-rabbit Alexa Flour 555 (1:500, Thermo Fisher Scientific, Cat# A-31572, RRID:AB_162543), donkey polyclonal anti-goat Alexa Flour 647 (1:500, Thermo Fisher Scientific, Cat# A-21447, RRID:AB_2535864), and goat polyclonal anti-chicken Alexa Flour 647 (1:500, Thermo Fisher Scientific, Cat# A32933, RRID:AB_2762845). Sections were washed twice with PBS (Gibco, Cat# 10,010,023) and then incubated with DAPI (1:500, Thermo Scientific, Cat# 62,248) for 5 min and mounted on glass slides using Fluorescence Mounting Medium (Agilent, Cat# 302,380–2). Confocal imaging was performed on a LSM900-Airy confocal microscope (Zeiss, Oberkochen, Germany) using a 20X objective. For each organoid section, 5 Z-stack images were captured at random locations along the periphery in order to avoid the necrotic core, using DAPI as guide for selecting regions of interest.

### Image analyses

Maximum intensity projections of Z-stacks were created using Fiji version 2.14.0 for Mac OS.^36^ A custom image analysis pipeline was optimized to classify and measure cells according to the expression of the selected markers using CellProfiler version 4.2.7 for Mac OS.^37^ First, modules were set up to identify and segment DAPI + objects as nuclei, neural progenitor cells by relating SOX2 + objects to nuclei, neurons by relating NeuN + objects to nuclei, oligodendrocyte lineage cells by relating OLIG2 + objects to nuclei, and astrocytes by relating GFAP + objects to nuclei. Then, the related objects were expressed as a fraction of total nuclei. Nuclear fluorescence intensity of GFAP, γH2AX, and CASP3 was then measured. Astrocytic area was calculated by dividing the area occupied by GFAP fluorescence with the number of GFAP + nuclei. Three samples in the vehicle condition, for which astrocytic area was artificially high due to only one GFAP + nuclei being detected, were removed as outliers. Finally, the number of γH2AX + and CASP3 + nuclei were calculated by thresholding of γH2AX and CASP3 nuclear fluorescence intensity, respectively, and subsequently expressed as a fraction of total nuclei.

### Statistical analyses

Statistical analyses were performed in GraphPad Prism 10 version 10.4.0 for Mac OS (GraphPad Software, Boston, Massachusetts USA, www.graphpad.com). Between-group differences were analyzed using the Mann–Whitney *U* test. All reported *p* values are two-tailed and *p* values < 0.05 were considered significant.

## Supplementary Information

Supplementary file 1 (PDF 724 kb)

## Data Availability

The data generated and analyzed during the current study are available from the corresponding author on reasonable request.
