# Supplementary material for: Clonidine prevents radiation-induced cell death in human brain organoids
Source: Sci Rep. 2025 Oct 31;15:38113. doi: 10.1038/s41598-025-26170-2 (PMC12579207; doi:10.1038/s41598-025-26170-2)
Supplement: Supplementary file 1 — (PDF 724 kb) [file 41598_2025_26170_MOESM1_ESM.pdf]

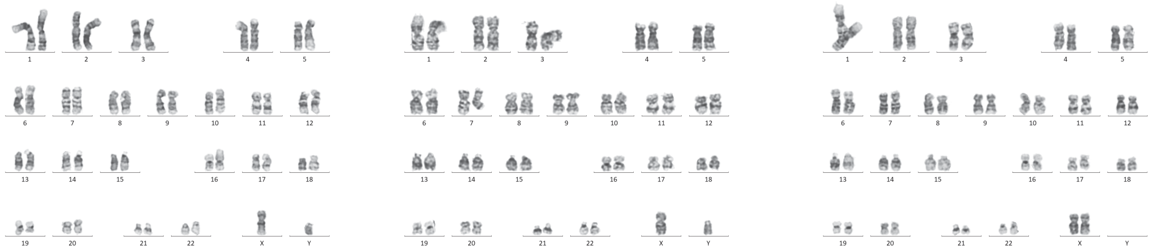

Supplementary Figure S1: Karyotyping of iPSC lines.

From left to right, karyograms for subjects S1 (male), S2 (male), and S3 (female).
